# Towards a HPV Vaccine Knowledgebase for Patient Education Content

**Published:** 2016

**Authors:** Dennis WANG, Rachel CUNNINGHAM, Julie BOOM, Muhammad AMITH, Cui TAO

**Affiliations:** a University of Texas at Austin; b Immunization Project, Texas Children’s Hospital; c University of Texas Health Science Center

**Keywords:** Ontology, Vaccine, Human Papillomavirus, Knowledgebase, Patient education

## Abstract

Human papillomavirus is a widespread sexually transmitted infection that can be prevented with vaccination. However, HPV vaccination rates in the United States are disappointingly low. This paper will introduce a patient oriented web ontology intended to provide an interactive way to educate patients about HPV and the HPV vaccine that will to empower patients to make the right vaccination decision. The information gathered for this initial draft of the ontology was primarily taken from the Centers for Disease Control and Prevention’s Vaccine Information Statements. The ontology currently consists of 160 triples, 141 classes, 52 properties and 55 individuals. For future iterations, we aim to incorporate more information as well as obtain subject matter expert feedback to improve the overall quality of the ontology.

## 1. Introduction

Human papillomavirus (HPV) is the most common sexually transmitted infection in the United States and currently affects 79 million individuals [3]. There are more than 120 types of HPV [11], some of which are considered high risk and can cause many types of cancers. HPV is spread through the skin and mucous membranes during direct skin-to-skin contact.

Fortunately, the HPV vaccine is an effective tool that can prevent the most common types of HPV infection, which can lead to several types of cancer. Studies have demonstrated that vaccinating young children before they are sexually active produces the strongest immune response and offers the best protection [1]. According to the Centers for Disease Control and Prevention (CDC), HPV vaccine coverage throughout the United States has been dismal compared to other vaccines recommended at the same age, in large part due to a lack of parental understanding about the disease and vaccine [13].

In today’s age of information and technology, patients are offered many options to educate themselves about vaccines. One study indicated that regardless of the accuracy of information, 70% of people turn towards Internet-based sources to retrieve knowledge about vaccines [19]. Other studies have pointed at the prevalence of anti-vaccination content on the web [15; 22]. While it is beneficial for health care providers to communicate to patients directly [20; 27], time constraints can limit and sometimes prohibit these interactions [17; 23]. Most clinics provide printed vaccine educational materials such as brochures, handouts, and booklets for patients to educate themselves [4]. Moreover, federal law requires that healthcare providers give a copy of the current Vaccine Information Statement (VIS) to all patients prior to vaccination. However, the reading levels of parents many times are often below that of the VIS and other patient materials[16; 18; 25], and patients sometimes ignore or do not receive adequate understanding from this kind of inactive learning approach to make critical vaccination decisions [28].

For this project, we propose the use of an ontological knowledgebase that stores patient-focused vaccine information. An ontology can semantically represent domain knowledge in a machine-understandable format, which enables automatic and intelligent queries. Our knowledgebase can represent complex vaccination information and make it scalable and query-able to deliver rich vaccine information as needed. In addition, the information is represented in a patient-friendly format with limited medical jargon so that patients can better understand the information [21]. Moreover, an ontology for patients creates opportunities for more interactive and precise delivery of vaccine information using patient-friendly natural language queries, where patients and parents receive direct and personalized answers, like VAMATA, a “Siri-like” application for online medical assistance in the military setting [26] or for a prototype interactive mobile application for vaccine education [9]. Therefore, we believe that a HPV ontology can serve as a foundation for an application that will empower patients to make vaccination decisions.

The Vaccine Information Statement Ontology (VISO) is an already existing ontology knowledgebase that contains defined classes and relationships pertaining to vaccines and their pathogens [8]. Using the developed VISO framework, our objective is to define HPV knowledge that is machine-readable and semantically-rich so that it can be incorporated into an interactive learning application for patients.

## 2. Methods

HPV vaccine information was collected from the VISs from the CDC website [1; 2; 6]. Supplemental information was also gathered from Offit and Moser’s *Vaccines and Your Child* handbook [24]. Sentences relevant to HPV, and its vaccine were parsed into simple statements called triples, consisting of a subject, predicate, and object. Using the VISO ontology specification, we incorporated the knowledge triples gathered from the sources into the ontology by either using an existing relationship within VISO or creating new ones to better include and represent information. The program we used to construct the ontology is Protégé 5.0, a commonly acknowledged, open-source software for ontology authoring. The ontology is represented using the Web Ontology language (OWL), which is a standard ontology language. The ontology was evaluated using the Semiotic Evaluation Management System (SEMS). SEMS is a prototype web-based tool that rates an ontology based on different aspects that factor into the overall score [12]. The main qualities that were assessed are *syntactic*, *pragmatic*, and *semantic*. The *syntactic* quality rates the ontology’s machine-readability, the *semantic* quality measures the usefulness of the ontology based on the terms’ *ambiguity* and *consistency*, and *pragmatic* measures the extensiveness of the ontology. Based on the evaluation result, the ontology can be further improved with several additional iterations.

## 3. Results

The ontology uses the previously developed VISO representation to define the conceptual class level, but also includes some refinement to accommodate complex HPV vaccine knowledge. The current version of the Vaccine Information Statement Ontology For Human Papillomavirus (VISO for HPV [7], and figure [5]) contains 160 triples, 141 classes (125 subclasses), 52 properties (36 object properties and 16 data properties), and 55 individuals.

We also obtained quality metrics using the semiotic metric evaluation from [10]. Using the SEMS web-based tool, we automatically generated the *syntactic*, *pragmatic*, and *semantic* scores. Table 1 outlines the quality score breakdown.

The overall quality score based on the three quality aspects amounted to 0.73. The *syntactic* score is 0.76 and is comprised of *lawfulness* and *richness* qualities, which are 1.00 and 0.51, respectively. *Semantic* aspect of the ontology scored 0.93. The *interpretability*, *consistency*, and *clarity* qualities make up the *semantic* aspect, which ranks, 0.88, 0.97, and 0.96, respectively. The *pragmatic* aspect score only included one factor, *comprehensiveness*, so its score is 0.50. Currently, the SEMS tool is still under development, and the *accuracy* quality (an aspect of the *pragmatic* quality) could not be calculated.

## 4. Discussion

Based on the analysis of the scores, our initial HPV ontology proves promising. The *syntactic* score (0.76) demonstrates that the high machine readability of the ontology due to correct use of syntax (*lawfulness*), and lack of total utilization of OWL features (*richness*). However, for this specific ontology and its use-case of only representing vaccine patient knowledge, some of the features may not be necessary, but as we continually develop VISO for HPV, there may be knowledge that may require other OWL features to better model the vaccine information. The *semantic* quality is also relatively high (0.93) resulting from low use of repetitive terms (*consistency*) and low use of ambiguous terms (*clarity*). Overall, the scores are quite promising for initial work, but evaluating the *accuracy* and expanding the use of OWL-based features are needed to fully evaluate the quality.

Creating the ontology posed many challenges. Even though there is already an existing VISO representation from which the HPV ontology is based, a few concept classes and subclasses had to be created or modified in order to better represent some knowledge triples. For example, a subclass called “*Adjuvant*” was conceptualized to describe specific ingredients added to vaccines, like monophosphoryl lipid A which is added to Cervarix, one of the licensed HPV vaccines. Another example, the subclass “*DNA Virus*” to describe Human Papillomavirus (HPV) was also created to elaborate on the type of virus that the HPV vaccines target. Another difficulty that we experienced was determining whether to include some of the information found pertaining to HPV in the ontology such as side effects, contraindications, and mechanism of action. Ultimately, any information determined to be beneficial and helpful to the patient was included. Additionally, some of the medical terms in the VIS documents are subject to interpretation and could be ambiguous to patients, such as the terms “*mildly ill*” versus “*moderately ill*”. Most patients will be unable to discern the difference between the degrees of illness; therefore, it can become misleading to patients.

By attaining a functional knowledgebase, we can potentially power the intelligence behind patient-centered interactive agents – mobile devices, kiosks, etc. – to improve patient vaccine literacy and address patient questions in which patient-provider interaction is lacking or could be improved. As for the *accuracy* quality, we will need to obtain feedback from subject matter experts to improve the overall quality once the prototype SEMS tool can facilitate *accuracy* evaluation for subject matter experts to rate. We are investigating the possibility of growing the knowledgebase with any information that is lacking; for example, inclusion of additional patient-level education about the cancers caused by HPV, such as cervical and oropharyngeal cancer. While the initial knowledgebase included basic information regarding HPV-related cancers, additional information is needed to improve the robustness of the knowledgebase. Encoding extended information like these into the knowledgebase can help raise awareness for the need of HPV vaccination from a cancer-awareness perspective. A neglected aspect, yet of great interest to us, is to go beyond encoding facts and information about HPV vaccine and consider incorporating multimedia content to enrich the knowledgebase. Ontological knowledgebases could indeed link the various concepts with complementary multimedia content, like video and images, to deliver dynamic information through the patient-centered interactive agents. In addition, there is also evidence that storytelling may have an impact to educate and raise awareness of vaccination [14]. Whether it would be specific stories linked to certain vaccine concepts or a method to semi-automatically generate a story, the implementation of storytelling components in a machine-readable knowledgebase is of interest and will be further explored.

## Figures and Tables

**Table 1.** Ontology evaluation scores.

	Quality	Quality Score	
Syntactic			0.76
	Lawfulness	1.00	
	Richness	0.51	
Semantic			0.93
	Interpretability	0.88	
	Consistency	0.97	
	Clarity	0.98	
Pragmatic			0.50
	Comprehensiveness	0.50	
***Overall Score***			***0.73***
